# Rapid and Sensitive Inhibitor Screening Using Magnetically Modulated Biosensors

**DOI:** 10.3390/s21144814

**Published:** 2021-07-14

**Authors:** Shira Roth, Amos Danielli

**Affiliations:** Faculty of Engineering, The Institute of Nanotechnology and Advanced Materials, Bar-Ilan University, Max and Anna Webb Street, Ramat Gan 5290002, Israel; shiraro@biu.ac.il

**Keywords:** magnetically aggregated biosensors, inhibitor screening, SARS-CoV-2, spike protein, angiotensin-converting enzyme 2, neutralizing antibodies, small molecules

## Abstract

Inhibitor screening is an important tool for drug development, especially during the COVID-19 pandemic. The most used in vitro inhibitor screening tool is an enzyme-linked immunosorbent assay (ELISA). However, ELISA-based inhibitor screening is time consuming and has a limited dynamic range. Using fluorescently and magnetically modulated biosensors (MMB), we developed a rapid and sensitive inhibitor screening tool. This study demonstrates its performance by screening small molecules and neutralizing antibodies as potential inhibitors of the interaction between the spike protein 1 (S1) of the severe acute respiratory syndrome coronavirus 2 (SARS-CoV-2) and the angiotensin-converting enzyme 2 (ACE2) receptor. The MMB-based assay is highly sensitive, has minimal non-specific binding, and is much faster than the commonly used ELISA (2 h vs. 7–24 h). We anticipate that our method will lead to a remarkable advance in screening for new drug candidates.

## 1. Introduction

The outbreak of coronavirus disease in 2019 (COVID-19) started a race to develop drugs to treat severe acute respiratory syndrome coronavirus 2 (SARS-CoV-2). Such drug development usually requires the identification of inhibitors (e.g., therapeutic antibodies, peptides, and small molecules) that inhibit the activity of the virus [1,2,3,4,5,6,7,8]. In general, a virus infection cycle includes several phases, such as adhesion, viral entry, endocytosis, replication, and virus release [3]. For example, in the early phase of the SARS-CoV-2 infection cycle, the receptor binding domain (RBD) of the spike protein 1 (S1) adheres to the host cell by binding to the angiotensin-converting enzyme 2 (ACE2) receptor [2,9,10]. Therefore, inhibiting the S1-ACE2 interaction may block the entry of the virus to the host cell, practically limiting the spread of the virus in the body [2]. Potential inhibitors of this interaction, including anti-S1 antibodies, anti-S1 peptides, nanosponges, and ACE2 inhibitors [3,11], can be used as therapeutic drugs or as neutralizing antibodies in neutralization and antigen assays.

Screening multiple potential inhibitors requires rapid and sensitive tools. The most commonly used in vitro screening tool is an enzyme-linked immunosorbent assay (ELISA) [12]. A 96-well plate is coated with an antibody or an antigen that specifically binds to the analyte of interest. Following multiple washing steps, a labeled detection antibody is added and binds to the analyte, forming a sandwich assay. The labeled antibody is usually detected using a colorimetric or chemiluminescence reaction. While an ELISA-based inhibitor screening assay is rather simple and enables high throughput, it is also laborious, time consuming, and relatively insensitive [13]. Other inhibitor screening assays that rely on magnetic beads (e.g., flow cytometry) are also used for the high throughput screening of inhibitors [14]. Because magnetic beads facilitate the separation steps, are well suited for automation, and improve assay sensitivity, magnetic beads-based assays are commonly used in various bioassays and for biosensing applications [15,16,17,18,19].

Previously, we introduced a novel platform that uses magnetically modulated biosensors (MMB) to rapidly detect very low concentrations of antibodies [20], proteins [21], and specific nucleic acid sequences [22,23,24]. We have shown that the MMB can rapidly identify protein-protein interactions (PPI) in vitro with very high sensitivity [25]. Here, we demonstrate for the first time the applicability of the biosensors for rapid and highly sensitive screening of PPI inhibitors. In particular, we screened potential S1-ACE2 inhibitors and showed that the MMB-based inhibitors screening assay can detect and quantitatively assess different types of inhibitors, such as neutralizing antibodies and small molecules. Overall, the MMB-based assay is much faster (less than two hours) than ELISA (~7–24 h) and has minimal non-specific binding. A simple, rapid, and cost-effective inhibitor screening assay can significantly reduce the time and cost of the first step in drug development—inhibitor selection—which is usually time consuming and laborious [26].

**Figure 1:**
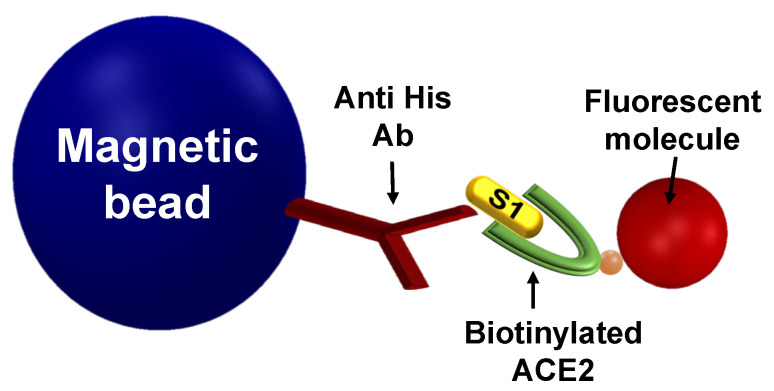
An MMB-based inhibitor screening assay of the S1-ACE2 interaction. Magnetic beads that are conjugated to anti-His antibodies are introduced to S1 recombinant protein. Then, biotinylated ACE2 protein is added and detected using a fluorescent molecule, such as streptavidin R-phycoerythrin (SA-PE).

**Figure A1:**
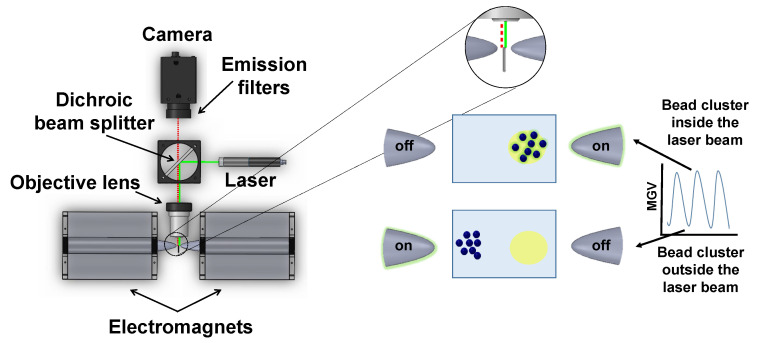
Schematic of laser beam system and data analysis.

In an MMB-based S1-ACE2 inhibitor screening assay (Figure 1), magnetic beads are attached to the S1 protein, and fluorescent molecules are attached to the ACE2 protein. Thus, when the proteins interact, the fluorescent molecules are connected to the magnetic beads. To increase the sensitivity of fluorescence detection, the magnetic beads with attached fluorescent molecules are aggregated using two opposing electromagnets and are transported from side to side, in and out of an orthogonal laser beam. When the beads pass through the laser beam, a fluorescence signal is emitted and collected using a digital camera (Figure A1).

**Figure 2:**
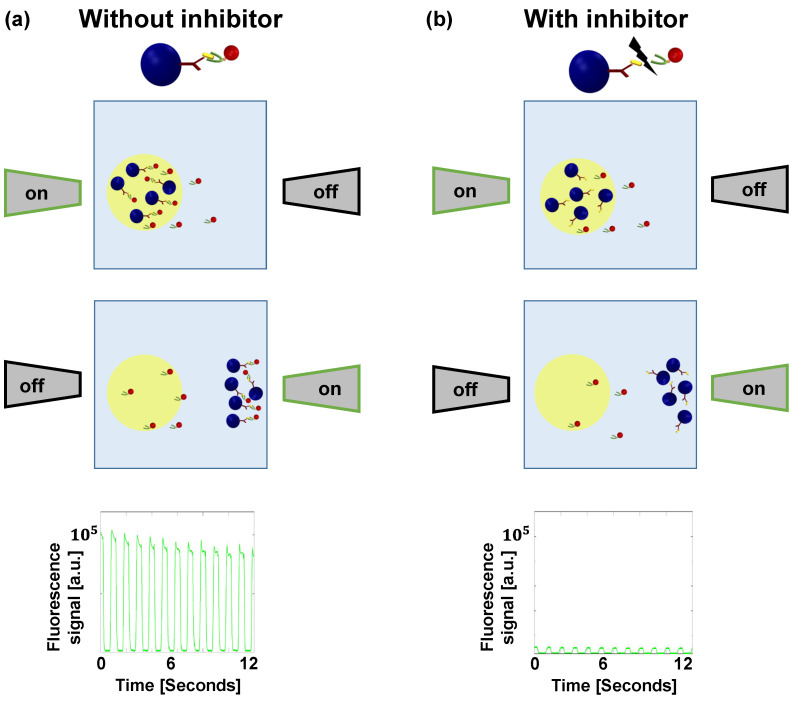
Illustration of magnetically modulated biosensors (MMB) with and without an inhibitor. (**a**) Two electromagnets, positioned on opposite sides of a sample cell, aggregate the magnetic beads and transport them in and out of an orthogonal laser beam. Without an inhibitor, the S1 protein interacts with the fluorescently labeled ACE2 receptor protein, and thus, when the magnetic beads pass through the laser beam, the fluorescence emission is high. Consequently, the average peak-to-peak signal is high. (**b**) When an inhibitor interferes with the interaction, the magnetic beads are not attached to the fluorescently labeled ACE2, and therefore, when the beads pass through the laser beam, the fluorescence emission is low. Consequently, the average peak-to-peak signal is small.

Without an inhibitor, the S1 protein interacts with the fluorescently labeled ACE2 receptor protein, and therefore, when the magnetic beads pass through the laser beam, the fluorescence emission is high. Consequently, the average peak-to-peak signal is high (Figure 2a). However, when an inhibitor interferes with the interaction, the magnetic beads are not attached to the fluorescently labeled ACE2. Therefore, when the beads pass through the laser beam, the fluorescence emission is low, and the average peak-to-peak signal is low, suggesting that a potential inhibitor of interest has been identified (Figure 2b).

## 2. Materials and Methods

### 2.1. Detection of the S1-ACE2 Interaction

To evaluate the MMB-based assay’s ability to detect the S1-ACE2 interaction and to determine its LoD, dynamic range, and analytical sensitivity, we coated tosylactivated magnetic beads (0.5 mg, Dynabeads M-280, 14203, Thermo Fisher Scientific, Waltham, MA, USA) with anti His antibodies (10 µg, 70796-3, Novagen, Madison, WI, USA) according to the manufacturer’s protocol, and photobleached them for ~20 h [27]. The conjugated magnetic beads (~$1.2\;\cdot \;10^{6}$ beads) were then mixed with a recombinant S1 protein (1.2 µg, S1N-C52H3 (His tag), Acro Biosystems, Newark, CA, USA) and incubated overnight at 4 °C. The conjugated beads were then washed by placing the samples on a MagJET separation rack (MR02, Thermo Fisher Scientific) for 2 min, taking out the solution, and pipetting the beads with 1 mL of a Tris buffer (50 mM Tris-HCl, 150 mM NaCl, pH 7.4, 1% *w/v* BSA, 0.05% *v/v* Tween-20). The washed beads were placed again on the separation rack, and the buffer was replaced with a new one. Following the single wash, the conjugated magnetic beads with the attached S1 protein were divided into a 96-well plate, with ~30,000 beads per well. Then, the buffer was taken out, and each sample was mixed with 100 µL of increasing concentrations of biotinylated ACE2 (AC2-H82F9, Acro Biosystems), ranging between a final concentration of 0.2 ng/mL and 2500 ng/mL, for one hour at 37 °C on a rotator.

**Figure 3:**
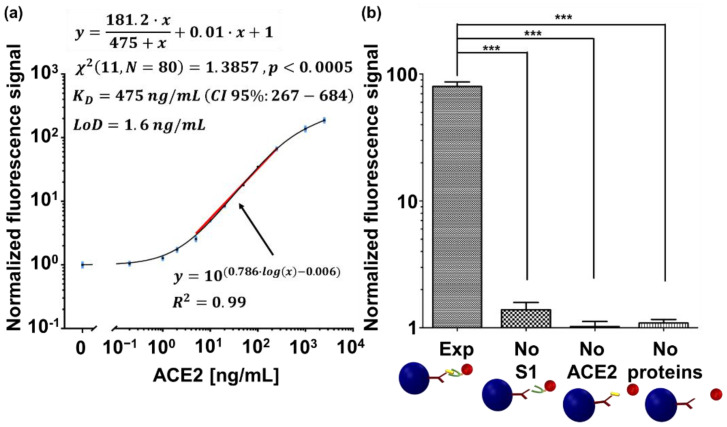
Detection of the S1-ACE2 interaction. (**a**) A dose response curve of S1 interaction with ACE2, measured by MMB. All measurements were normalized to the average signal of the conjugated beads. Using all the replicates, the curve was fitted to one site-total binding model. The extracted $K_{D}$ is reported as $K_{D}$ (CI 95%: lower limit–upper limit). The chi-square Goodness of fit is reported as $\chi^{2}$ (degrees of freedom, sample size). The solid red line was fitted using a non-linear regression analysis to the log-log dose response curve, and it represents the analytical sensitivity of the assay. Error bars (in blue) represent the standard error of the mean (SEM) of blank measurements (n=5) and all other concentrations (5≤n≤8). (**b**) Negative controls to the dose response. Abbreviations: “Exp”, conjugated magnetic beads with the attached S1 protein are introduced to 250 ng/mL ACE2 protein and SA-PE. “No S1”, conjugated magnetic beads without the attached S1 protein are introduced to 250 ng/mL ACE2 and SA-PE. “No ACE2”, conjugated magnetic beads with the attached S1 protein are introduced to SA-PE. “No proteins”, conjugated magnetic beads are introduced to SA-PE. Three asterisks (***) indicate a statistical significance of p<0.001. All measurements were normalized to the signal of the conjugated magnetic beads. The normalized fluorescence signal values are presented as the mean ± SEM.

To evaluate the possible contribution of non-specific binding to the MMB signal, we compared a positive sample to three negative controls. The positive experiment (“Exp” in Figure 3b) included ~30,000 anti-His conjugated magnetic beads per well, with the attached S1 protein that were introduced to 100 µL of biotinylated ACE2 at a concentration of 250 ng/mL of biotinylated ACE2. The first negative control (“No S1” in Figure 3b) included ~30,000 anti-His conjugated magnetic beads per well, without the attached S1 protein that were introduced to 100 µL of biotinylated ACE2 at a concentration of 250 ng/mL. The second negative control (“No ACE2” in Figure 3b) included ~30,000 anti-His conjugated magnetic beads with the attached S1 protein that were introduced to Tris buffer instead of the biotinylated ACE2. The third negative control (marked in Figure 3b as “No proteins”) included ~30,000 anti-His conjugated magnetic beads per well that were not introduced to either S1 or biotinylated ACE2 proteins.

All samples, including the negative controls, were then washed once by placing the 96-well plate on a magnetic 96-well separator (A14179, Thermo Fisher Scientific) for 2 min, taking out the solution and pipetting the beads with 200 µL of the Tris buffer. The washed beads were then placed again on the magnetic 96-well separator for 2 min, and the solution was taken out. Then, the complexes were incubated for 20 min at room temperature with 100 µL of 1 µg/mL of streptavidin *R-phycoerythrin* (SA-PE, PJRS20-1, Agilent Technologies, Santa Clara, CA, USA). Subsequently, after a single buffer replacement to remove unbound fluorescent molecules, 100 µL of the final solution was loaded into a borosilicate glass cuvette and measured in the MMB system [25]. The LoD was calculated as three standard deviations over the blank measurement. To find the analytical sensitivity of the assay, which is the slope of the dose response [28], the curve was fitted using a non-linear regression analysis to a log-log line, using GraphPad. The statistical analysis in Figure 3b was performed using a one-way ANOVA and Dunnette’s post-hoc multiple comparison test (3≤n≤9).

### 2.2. Inhibition of the S1-ACE2 Interaction

**Figure 4:**
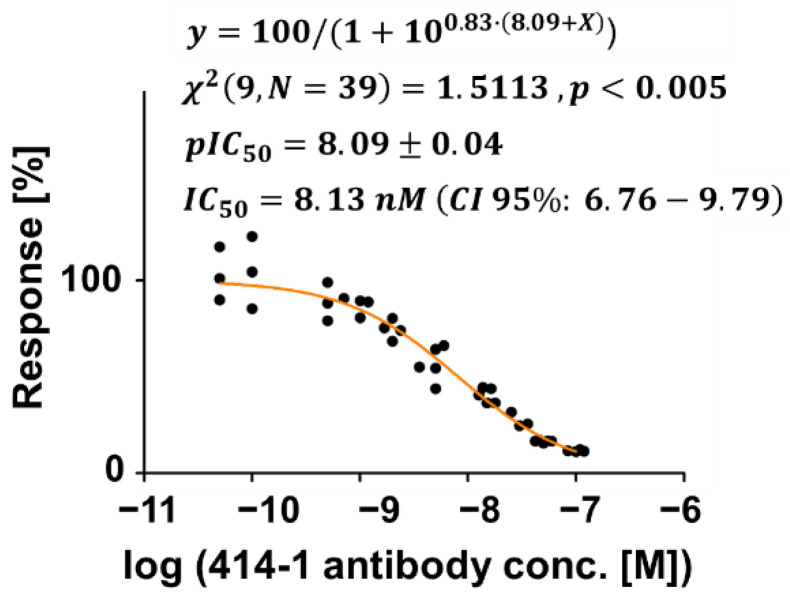
Assessing an anti-S1 antibody (414-1) as an inhibitor of the S1-ACE2 interaction. Binding curve of the S1-ACE2 interaction with increasing concentrations of the anti-S1 antibody. Measurements were normalized to the average signal (designated as 100% Response) when S1 binds with ACE2, at 0 nM of the anti-S1 antibody. The orange line was fitted using a non-linear regression analysis to the log (inhibitor) versus the normalized response using GraphPad. The chi-square Goodness of fit is reported as $\chi^{2}$ (degrees of freedom, sample size). IC_50_ and pIC_50_, and their SEM were extracted from the fitted line and are reported as ${\mathrm{pIC}}_{50}\;\pm \;\mathrm{SEM}$ and ${\mathrm{IC}}_{50}$ (CI 95%: lower limit–upper limit). For each concentration 3≤n≤5.

We evaluated the MMB-based S1-ACE2 inhibitor screening assay by testing neutralizing antibodies and small molecules. To demonstrate that the MMB-based assay can screen neutralizing antibodies as potential inhibitors, we chose the fully validated and experimentally demonstrated anti-S1 antibody (clone 414-1). This antibody had the best neutralizing effect amongst the 11 neutralizing antibodies found by Wan et al. [2], and its inhibitory effect has already been validated using ELISA [2,29]. To assess the inhibition of the S1-ACE2 interaction by the anti-S1 antibody, the conjugated magnetic beads with the attached S1 protein were incubated for one hour at 37 °C on a rotator with 50 µL of 2000 ng/mL of the biotinylated ACE2 protein and 50 µL of increasing concentrations of anti-S1 antibody (AM001414, Active motif, Carlsbad, CA, USA), ranging between a final concentration of 0.05 nM and 100 nM (Figure 4).

**Figure 5:**
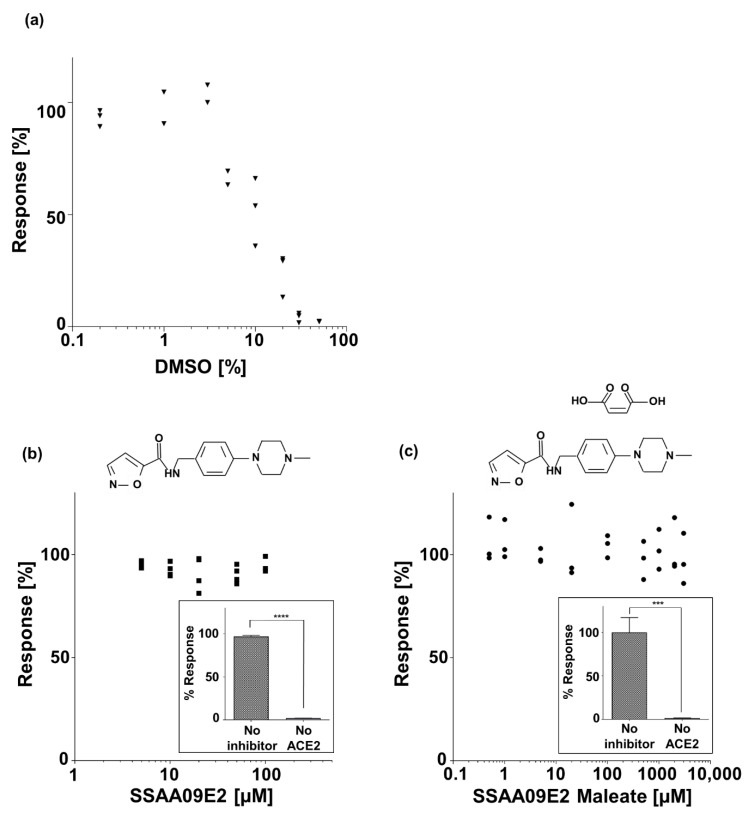
Assessing a small molecule (SSAA09E2) as an inhibitor of the S1-ACE2 interaction. In each binding curve, the signals were normalized to the signal at 0 µM of inhibitor, which was set as 100% Response. (**a**) A binding curve of S1-ACE2 interaction with increasing concentrations of DMSO. The experiment was repeated three times (n=3). (**b**) A binding curve of S1-ACE2 interaction with increasing concentrations of SSAA09E2 in a final concentration of 1% *v/v* DMSO. The experiment was repeated three times (n=3). (Inset) Abbreviations: “No inhibitor”, conjugated magnetic beads with the attached S1 protein were incubated with biotinylated ACE2 protein and SA-PE. “No ACE2”, conjugated magnetic beads with the attached S1 protein were incubated with the inhibitor and SA-PE, but without the ACE2 protein. Measurements were normalized to the signal (designated as 100% Response) when S1 binds with ACE2 without an inhibitor and without DMSO. Error bars represent the standard error of the mean value of four experiments (n=4). Four asterisks (****) indicate a statistical significance of p<0.0001. (**c**) A binding curve of S1-ACE2 interaction with increasing concentrations of SSAA09E2 Maleate dissolved in DDW. The experiment was repeated three times (n=3). (Inset) Abbreviations: “No inhibitor”, conjugated magnetic beads with the attached S1 protein were incubated with biotinylated ACE2 protein and SA-PE. “No ACE2”, conjugated magnetic beads with the attached S1 protein were incubated with the inhibitor and SA-PE, but without the ACE2 protein. Measurements were normalized to the average signal (designated as 100% Response) when S1 binds with ACE2 without an inhibitor. Error bars represent the standard error of the mean value of five experiments (n=5). Three asterisks (***) indicate a statistical significance of p<0.001.

To assess the inhibition of S1-ACE2 interaction by a small molecule, we selected the N-[[4-(4-Methyl-1-piperazinyl)phenyl]methyl]-5-isoxazolecarboxamide (SSAA09E2) molecule, which was previously demonstrated as an inhibitor for the interaction between SARS-S RBD and ACE2 [30], and therefore, was also suggested as an inhibitor of the SARS-CoV-2 [31]. According to the manufacturer’s recommendation, the small molecule SSAA09E2 (GLXC-03528, Glixx Laboratories Inc., Hopkinton, MA, USA) is dissolved in dimethyl sulfoxide (DMSO) at a concentration of 3 mg/mL, which is less than its reported solubility (~8 mg/mL). Because DMSO itself is known to have an inhibitory effect on enzymes [31], we first evaluated possible inhibition of the interaction between S1 and ACE2 by DMSO. The conjugated magnetic beads with the attached S1 protein were incubated for one hour at 37 °C on a rotator with 20 ng/mL of the biotinylated ACE2 protein in increasing final concentrations of DMSO ranging between 0.2% *v/v* and 50% *v/v* (Figure 5a). DMSO dilutions were performed using Tris buffer.

To evaluate the inhibition effect of the SSAA09E2 on the interaction between the S1 protein and ACE2 receptor, the conjugated magnetic beads with the attached S1 protein were incubated for one hour at 37 °C on a rotator with 20 ng/mL of the biotinylated ACE2 protein and increasing concentrations of SSAA09E2 ranging between a final concentration of 5 µM and 100 µM, in a final concentration of 1% *v/v* DMSO (Figure 5b). To test whether the SSAA09E2 molecule binds non-specifically to the S1 protein or to the conjugated magnetic beads, the conjugated magnetic beads with the attached S1 protein were incubated with 100 µM of SSAA09E2, but without the ACE2 protein (inset of Figure 5b). The means of “No inhibitor” and “No ACE2” were compared using an unpaired t-test (two tailed) with a CI of 95%.

To extend the demonstration using other solvents and concentrations, we also tested the inhibition effect of the Maleate salt form of the SSAA09E2, N-[[4-(4-Methyl-1-piperazinyl)phenyl]methyl]-5-isoxazolecarboxamide Maleate (GLXC-22577, Glixx Laboratories Inc.). The conjugated magnetic beads with the attached S1 protein were incubated for one hour at 37 °C on a rotator with 20 ng/mL of the biotinylated ACE2 protein and increasing concentrations of SSAA09E2 Maleate ranging between a final concentration of 0.02 mM and 5 mM (Figure 5c). To test whether SSAA09E2 Maleate binds non-specifically to the S1 protein or to the conjugated magnetic beads, the conjugated magnetic beads with the attached S1 protein were incubated with 1 mM of SSAA09E2 Maleate, but without the ACE2 protein (inset of Figure 5c). The means of “No inhibitor” and “No ACE2” were compared using an unpaired t-test (two tailed) with a CI of 95%.

All samples, including the negative controls, were washed once with the Tris buffer and incubated at room temperature for 20 min with 100 µL of 1 µg/mL SA-PE. Finally, after a single buffer replacement, 100 µL of the final solution was loaded into a borosilicate glass cuvette and measured in the MMB system [25].

## 3. Results

### 3.1. Detection of the S1-ACE2 Interaction

The dose response of the interaction between S1 and ACE2, shown in Figure 3a, follows a sigmoidal binding curve, with a calculated limit of detection (LoD) of 1.6 ng/mL and an over 4-log dynamic range. The analytical sensitivity is 0.786 (normalized fluorescence signal/[ng/mL]), and the coefficient of variance (CV) is less than 18% over the entire range. The curve was fitted using GraphPad to one site-total binding model (See Appendix A). The extracted $K_{D}$ was 475 ng/mL. To avoid confusion, the dynamic range is defined as the ratio between the largest and smallest concentrations for which a signal is detected, the analytical sensitivity is defined as the ability of an analytical procedure to produce a change in signal for a defined change in the quantity being measured, and the LoD is defined as the minimal number of target molecules that can reliably be detected and differentiated from the blank measurement [28]. When compared to the negative controls (Figure 3b), the normalized signal of the experiment (“Exp”) is significantly higher (p<0.001) than the normalized signal of the negative controls (“No S1”, “No ACE2”, and “No proteins”).

### 3.2. Assessing an Anti-S1 Antibody as an Inhibitor of the S1-ACE2 Interaction

Figure 4 demonstrates the inhibitory effect of the anti-S1 antibody on the interaction between S1 and ACE2. The inhibitory binding curve was fitted using GraphPad to a log(inhibitor) versus normalized slope-variable slope model (See Appendix A). The half maximal inhibitory concentration (IC_50_) was calculated to be 8.13 nM (CI 95%: 6.76–9.79), and the CV for each concentration is less than 18% over the entire range. The z factor, which is a measure of an assay’s ability to identify inhibitors [32], was calculated to be 0.9 (Appendix A).

### 3.3. Assessing a Small Molecule (SSAA09E2) as an Inhibitor of the S1-ACE2 Interaction

The effect of DMSO concentration on the interaction between S1 and ACE2 is presented in Figure 5a. An inhibitory effect begins at a DMSO concentration of 5% *v/v*, reaching an inhibition of ~98% at 50% *v/v.* The effect of both SSAA09E2 (in 1% *v/v* DMSO) and SSAA09E2 Maleate (dissolved in DDW) on the interaction between S1 and ACE2 is shown in Figure 5b,c. The response relative to zero concentration of the inhibitors remains constant (~100%) at all concentrations. At each concentration of the SSAA09E2 and SSAA09E2 Maleate, the CV is less than 17% and 26%, respectively. The insets of Figure 5b,c evaluate possible non-specific binding of SSAA09E2 and SSAA09E2 Maleate to the S1 protein or to the conjugated magnetic beads. The response of the sample without ACE2 is significantly lower than the positive control (i.e., no inhibitor) for both SSAA09E2 (p<0.0001) and SSAA09E2 Maleate (p<0.001).

## 4. Discussion

In drug development, the most commonly used inhibitor screening tool is an ELISA test. However, ELISA-based inhibitor screening is time consuming and has a limited dynamic range. For example, according to the manufacturer’s protocol, the 96-well plate used in ELISA should be coated with the relevant proteins and incubated overnight a day before the experiment. In addition, the ELISA protocol includes several incubation stages and at least four sets of multiple washing steps between them, extending the overall assay time to ~24 h. In comparison, the modulation of the signal in MMB eliminates the need for washing and separation steps, and thereby shortens and simplifies the detection protocol. The total turnaround time of the MMB-based assay is less than 2 h. Moreover, in the MMB-based inhibitor screening assay, the magnetic beads can be pre-conjugated with the antibodies and the S1 protein, and then stored for future use (Figure S1, Supporting Information). Nevertheless, to apply this method to investigate other inhibitor screening assays, conjugation with different antibodies and proteins will require verification of the pre-conjugated magnetic beads’ stability. While it might be possible to store the ELISA plates after coating, the biological activity of the coated plates after storage should also be verified. Without the overnight incubation, the total turnaround time of the ELISA is ~7 h. It should be noted that in ELISA, the reaction and readout occur on a 2D surface. However, in the MMB-based assay, the beads are floating and continuously mixed, and therefore, the reaction and readout take place on a 3D surface. Hence, a lower amount of protein can be used in the MMB (~0.03 µg/well) versus ELISA (0.2 µg/well) [33].

When plotting a saturation curve on a logarithmic scale, a sigmoidal shape of the binding curve (Figure 3) is expected [34,35], and was previously reported in the case of S1-ACE2 interaction using recombinant proteins in vitro [8,36]. Using the MMB-based inhibitor screening assay, the half maximal inhibitory concentration (IC_50_) of the anti-S1 antibody was calculated to be 8.13 nM (CI 95%: 6.76–9.79), which is on par with the results achieved by the manufacturer in a neutralization test (15.77 nM) [29] and by Wan et al. in both neutralization and ELISA tests (1.75 nM and 2.96 nM, respectively) [2]. In addition, the z factor was calculated to be 0.9, which, according to Zhang et al., means that the separation between the highest concentration and the blank measurement is significant, and the assay is excellent in relation to screening [32]. Moreover, the calculated LoD in the S1-ACE2 binding assay (1.6 ng/mL) is similar to those reported by commercially available ELISA kits, but is achieved with a higher dynamic range (4-log vs. 2-log) [33,37]. For example, an ELISA-based inhibitor assay using SARS-CoV-2 S1 protein can bind Human ACE2 (Fc tag) with an LoD of ~0.2 ng/mL [33]. When detected by Streptavidin-HRP [37], immobilized SARS-CoV-2 S protein RBD can bind human ACE2-Biotin with an LoD of ~1 ng/mL. In ELISA, due to the enzymatic reaction, the signal reaches saturation following a slight change of target concentration. Hence, the analytical sensitivity is high, but the dynamic range is limited. It should be noted that the dynamic range of a fluorescence-based ELISA may be broader. In MMB, the magnitude of the detected fluorescence signal is proportional to the number of target molecules in the sample [21,25], and therefore, MMB provides quantitative results and a larger dynamic range.

To calculate the $K_{D}$ using MMB, we fitted the MMB data to one site-total binding model. This model was selected based on a 1:1 binding of S1 to the ACE2 receptor [10,38,39,40]. The model considers the total binding, including specific and non-specific binding. It should be noted that this analysis assumes that only a small fraction of ACE2 binds, which means that the concentration added is virtually identical to the free concentration. In our setup, we cannot verify this assumption or use another model that considers ligand depletion, which is applicable to radioligands only. Therefore, we treated the calculated $K_{D}$ as empirical description of the data and not as the true $K_{D}$. According to this analysis, $K_{D}≅475\;\mathrm{ng}/\mathrm{mL}$, which, after dividing by the ACE2 molecular mass, equals to $K_{D}≅4.25\;\mathrm{nM}$. This dissociation constant is in the range of $K_{D}$ values reported by others (1.2–120 nM) for this interaction [10,38,39,40,41]. To calculate the $K_{D}$ using ELISA, one has to perform a modified ELISA that also includes an indirect ELISA to find the free concentration of ligand [42,43]. It should be noted that even the manufacturer of the S1 and ACE2 proteins based his $K_{D}$ measurements (21.4 nM) on Biolayer Interferometry (BLI) assay and not on ELISA [33]. Taking the mid-point of 15 ng/mL for the ELISA, results in a $K_{D}=0.13\;\mathrm{nM}$, which is more than two orders of magnitude lower than the manufacturer’s reported value via the BLI assay. This difference may occur because the enzymatic reaction in ELISA causes the signal to saturate quickly and not proportionally to the concentration of the ligand, making it erroneous to simply take the midpoint of the binding curve.

The significant difference between the normalized fluorescence signal of the experiment (Figure 3b) and the negative controls show that the MMB-based assay has minimal non-specific binding between the magnetic beads and either the fluorescent molecules or the receptor, or between the S1 protein and the fluorescent molecules. Furthermore, the slope of 0.01 (Normalized fluorescence signal/[ng/mL]) in the equation of the fitted dose response curve (Figure 3a) represents the non-specific binding of the assay. The apparent low non-specific binding of the assay contributes to its large dynamic range.

Due to the physical manipulation of the magnetic biosensors in the sample cell, they are continuously mixed, and therefore, whenever they pass the laser beam, different regions of their surface are exposed to light. The modulation averages out the background noise and minimizes the possible effect of photobleaching [27]. Thus, the average fluorescence signal reliably represents the number of fluorescent molecules attached to the beads. Nevertheless, at low inhibitor concentrations, it is possible that some of the complexes on the beads are inhibited, and some are not, resulting in inhomogeneity of the fluorescence signal from the magnetic beads. This inhomogeneity can explain the higher standard deviation of different experiments seen at low anti-S1 antibody concentrations (Figure 4).

Because SARS and SARS-CoV-2 have similar binding modes and interfaces between the ACE2 cell receptor and the RBD [10], it was assumed that inhibitors that hinder the interaction between ACE2 and the spike protein of SARS will also inhibit the interaction of S1-ACE2 in SARS-Cov-2 [3,6]. For example, although it has not been tested so far, the small molecule SSAA09E2 that was shown to inhibit the interaction between SARS-S-receptor binding domain (RBD) and ACE2 [30], was suggested as an inhibitor for SARS-CoV-2 [31]. Here, using the MMB-based inhibitor screening assay, we assessed the potential of this small molecule as an inhibitor of the S1-ACE2 interaction. Because SSAA09E2 is dissolved in DMSO, we tested whether DMSO itself inhibits the interaction between S1 and ACE2. DMSO concentrations of 1–60% *v/v* were previously reported to have an inhibitory effect on enzymes, such as steroid sulfatase, E. coli β-galactosidase, glutamate dehydrogenase, and E. coli phosphomonoesters [44]. Lower concentrations of DMSO, ranging between 0.06–1% *v/v*, were reported to have an inhibitory effect of 10.1–36.7% on human acetylcholinesterase, reaching 98% inhibition at a final DMSO concentration of 16.6% *v/v* [45]. However, inhibition of the interaction between S1 and ACE2 by DMSO has not been reported. Here, the inhibitory effect of DMSO is seen at concentrations of 5–50% *v/v* (Figure 5a). Currently, low concentrations of DMSO (0.3–0.5% *v/v*) [5,46] are commonly used as a negative control for SARS-CoV-2 inhibitor screening experiments [9,47]. Therefore, to test the inhibitory effect of the small molecule SSAA09E2 on the S1-ACE2 interaction, a constant low concentration of 1% *v/v* DMSO, which, according to our results, does not inhibit the interaction itself, was used. At all concentrations tested (Figure 5b), the response remained similar to the response without an inhibitor (100% response). While the inhibitory effect of SSAA09E2 on the interaction between SARS-S RBD and ACE2 was seen at an SSAA09E2 concentration of 5–20 µM [30], here, even at the highest SSAA09E2 concentration of 100 µM, no inhibition of the SARS-CoV-2 S1-ACE2 was seen. Because the SSAA09E2 molecule does not bind non-specifically to either the S1 protein, the fluorescent molecule, or to the conjugated magnetic beads (inset of Figure 5b), it can be deduced that the SSAA09E2 molecule does not inhibit the interaction between S1 protein and ACE2 receptor in vitro.

To extend the demonstration using other solvents and concentrations, we also tested the Maleate salt form of the SSAA09E2 molecule. The SSAA09E2 Maleate can be dissolved in an aqueous solution, enabling testing of higher inhibitor concentrations. At all concentrations tested—even at the highest inhibitor concentration of 5 mM—there is no decrease in binding (Figure 5c). Because the Maleate salt itself does not bind non-specifically to either the S1 protein, the fluorescent molecule, or the conjugated magnetic beads (inset of Figure 5c), it can be concluded that SSAA09E2 Maleate does not inhibit the interaction between S1 and ACE2 in vitro.

Because the MMB system requires simple optical components, it can be affordable to many research groups. However, implementing a new method in a laboratory requires extensive training and lab protocol adjustments. Moreover, while the MMB assay time is relatively short and simple, a limiting factor of the current MMB system is its low throughput. Compared with ELISA, which can run on a 96-well or 384-well plate simultaneously, the current MMB system can read a single sample at a time. Ongoing modification to the MMB system will enable simultaneous detection of 96 samples in a 96-well plate.

## 5. Conclusions

We present a rapid and quantitative MMB-based inhibitor screening assay that can detect and classify different types of molecules as inhibitors or non-inhibitors of the S1-ACE2 interaction. Compared with the commonly used ELISA, the MMB-based assay is much faster and has minimal non-specific binding. This assay can also be easily adjusted to other PPI inhibitor screening. Future work may include continuous measurements and screening of multiple inhibitors simultaneously using a cell-based system and clinical samples. We anticipate this method can be utilized as a global tool for inhibitor screening, specifically for identifying SARS-CoV-2 inhibitors as new drug candidates.

## Supplementary Materials

The following are available online at https://www.mdpi.com/article/10.3390/s21144814/s1, Figure S1: Assessing the functionality of the conjugated magnetic beads over time.

## Appendix A

### Appendix A.1. Magnetically Modulated Biosensors

A 3.5 mm diameter beam from a 532 nm laser diode module (CPS532, ThorLabs, Newton, NJ, USA), working at 0.25 mW, passes through two lenses (200 mm and 100 mm focal lengths, ThorLabs, not shown in the figure) and is diverted by a dichroic beam splitter (BrightLine Di02-R532, Semrock, Rochester, NY, USA) into an objective lens (M-10X, 0.25NA Newport, Irvine, CA, USA). The beam exiting the objective lens is focused on a rectangular borosilicate sample cell containing the magnetic beads, with inner dimensions of 8 mm × 0.4 mm × 70 mm (2548, Vitrocom, Mountain Lakes, NJ, USA). The emitted fluorescence is collected using the same objective lens, passes through the dichroic beam splitter and two emission filters (FF03-575/25-25, Semrock), and is detected by a CMOS camera (GS3-U3-23S6M-C, FLIR, Wilsonville, OR, USA). To avoid saturation, excitation and emission signals are attenuated by various ThorLabs optical density filters.

To increase the sensitivity of the fluorescence detection, two electromagnets, one on each side of the sample cell, induce oscillating magnetic field gradients at a frequency of 1 Hz that concentrate the beads from the entire solution into a small cluster in the detection area and transport them in and out of the laser beam. The modulation separates the fluorescence signal from the background noise. When the magnetic beads pass through the laser beam, a strong fluorescence signal is emitted from the molecules attached to the beads. When the magnetic beads are outside of the laser beam, only the constant background noise is detected, generated by the sample matrix or by unbound fluorescent molecules. For each sample, the camera acquires a sequence of 600 frames over a period of 12 s. The mean grey value from the laser beam area of each frame is integrated, and the peak-to-peak difference is calculated and averaged using Matlab (Figure A1).

### Appendix A.2. Z-Factor Calculation

According to ref [32], the z factor is usually calculated for assay quality assessment of high throughput screening assays and is defined as:

(A1) $$z=1-\frac{3\;\cdot \;SD\;of\;sample+3\;\cdot \;SD\;of\;control}{\left|mean\;of\;sample-mean\;of\;control\right|}$$

However, for a small sample set, a t-distribution model is usually used. Here, because the number of samples in the assay presented in Figure 4 was 3–5 (i.e., n<30), the z factor was calculated between the highest concentration and the blank measurement based on the t-distribution, with a 99% confidence interval:

(A2) $$z=1-\frac{t\;\cdot \;\left(SD\;of\;sample\right)/\surd n+t\;\cdot \;\left(SD\;ofcontrol\right)/\surd n}{\left|mean\;of\;sample-mean\;of\;control\right|}$$

where the degree of freedom was df=n−1.

### Appendix A.3. Curve Fitting

The binding curve (Figure 3a) was fitted using a one site-total binding model [48]:

(A3) $$y=\frac{B_{max}\;\cdot \;x}{K_{D}\;+\;x}\;+\;NS\;\cdot \;x\;+\;background$$

where:

$B_{max}$ is the maximum specific binding [normalized fluorescent signal],
$K_{D}$ is the equilibrium dissociation constant [ng/mL],
NS is the slope of nonspecific binding [normalized fluorescent signal/(ng/mL)],
background is the average normalized fluorescent signal measured without a fluorescent marker (i.e., 0 ng/mL of biotinylated ACE2 protein).

The Goodness of fit was calculated using the chi-square test.

The inhibitory binding curve (Figure 4) was fitted using a log(inhibitor) versus normalized response-variable slope model [49]:

(A4) $$y=100/\left(1\;+\;10^{\left(LogIC_{50}\;-\;x\right)*Hillslope}\right)$$

where:

$IC_{50}$ is the concentration of inhibitor that gives a response halfway between bottom and top,
Hill slope describes the steepness of the family of curves.

The Goodness of fit was calculated using the chi-square test.
