# Supplementary material for: Rapid and Sensitive Inhibitor Screening Using Magnetically Modulated Biosensors
Source: Sensors (Basel). 2021 Jul 14;21(14):4814. doi: 10.3390/s21144814 (PMC8309820; doi:10.3390/s21144814)
Supplement: Supplementary file 1 [file sensors-21-04814-s001.zip › sensors-1282327-supplementary.pdf]

Supplementary Materials

Rapid and sensitive inhibitor screening using magnetically modulated biosensors

Shira Roth, Amos Danielli*

Faculty of Engineering, The Institute of Nanotechnology and Advanced Materials, Bar-Ilan University, Max and Anna Webb Street, Ramat Gan, 5290002, Israel

*Email: amos.danielli@biu.ac.il

Content

Figure S1: Schematic of laser beam system and data analysis

Assessing the functionality of the conjugated magnetic beads over time

In the MMB-based inhibitor screening assay, the magnetic beads can be pre-conjugated with the antibodies and stored for future use. To evaluate the functionality of the pre-conjugated magnetic beads over time, we coated tosylactivated magnetic beads (0.5 mg, Dynabeads M-280, 14203, Thermo Fisher Scientific, Massachusetts, USA) with anti His antibodies (10 µg, 70796-3, Novagen, New Jersey, USA) according to the manufacturer's protocol. The conjugated magnetic beads (~$1.2 \cdot 10^6$ beads) were then mixed with a recombinant S1 protein (1.2 µg, S1N-C52H3, Acro Biosystems, Newark, USA) and incubated overnight at 4°C. The conjugated beads with the attached S1 protein were divided into two aliquots and stored at 4°C.

On day 0, after a single wash with a Tris buffer (50mM Tris, 150 mM NaCl, 1% BSA, 0.05% Tween-20, pH 7.4), the first aliquot of the conjugated magnetic beads with the attached S1 protein were divided into a 96-well plate, with ~30,000 beads per well. Then, the buffer was taken out and each sample was mixed with 100 µL of increasing concentrations of biotinylated ACE2 (AC2-H82F9, Acro Biosystems, Newark, USA), ranging between 2 ng/mL and 20 ng/mL, for one hour at 37°C on a rotator. All samples were then washed once with the Tris buffer, and the complexes were incubated for 20 minutes at room temperature with 100µL of 1 µg/ml of streptavidin R-phycoerythrin (SA-PE, PJRS20-1, Agilent Technologies, California, USA). Subsequently, after a single buffer replacement to remove unbound fluorescent molecules, 100 µL of the final solution was loaded into a borosilicate glass cuvette and measured in the MMB system. Seven days later, we repeated the same experiment using the second aliquot of the conjugated beads. No changes in the normalized fluorescence signal were observed between the two experiments (Figure S1).

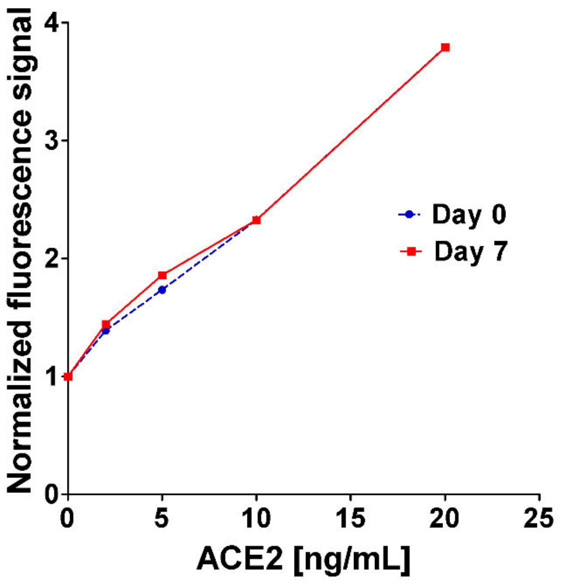

Figure S1. Assessing the functionality of the conjugated magnetic beads over time
